# A comprehensive geotechnical and geophysical assessment of the foundation sublayers in egypt’s new administrative capital

**DOI:** 10.1038/s41598-025-29246-1

**Published:** 2025-12-05

**Authors:** Mahmoud A. Abed, Adel A. A. Othman, Salah Shebl, Mahmoud Zayed, Mohamed H. Farag

**Affiliations:** 1 Geology Department, Faculty of Science, Al-Azhar University, Cairo, 11884 Egypt; 2 Geophysics Laboratory, Exploration Department, Egyptian Petroleum Research Institute, Cairo, Egypt; 3 EPRI Core Analysis Center, Egyptian Petroleum Research Institute (EPRI), Nasr City, Cairo Egypt

**Keywords:** Geotechnical, Geophysical, ERT, Shallow seismic refraction, New administrative capital, Engineering, Solid Earth sciences

## Abstract

This study presents a comprehensive geotechnical and geophysical characterization of foundation sublayers in Egypt’s New Administrative Capital, using thirteen Electrical resistivity Tomography (ERT) profiles with seismic velocity data from seventeen Shallow seismic refraction sites and three strategically selected Multichannel Analysis of Surface Waves (MASW) locations. The MASW sites were selected based on geoelectric profiles that reflect the complete types of lithologic variability across the study area, ensuring representative shear wave velocity (Vs) measurements. Resistivity results delineating four major subsurface units: a variable unit of sand, clay, and rock fragments; a limestone unit, a clay unit, and a sandstone unit. Seismic data enabled the calculation of key geotechnical parameters such as: rigidity modulus, Poisson’s ratio, Young’s modulus, and bulk modulus, revealing zones of high competence in the northeastern and northwestern parts, and incompetent materials in the central and southwestern parts of the study area and fairy to moderately competence between them. Material competence was assessed using the concentration index, material index, and stress ratio, which collectively divided the area into zones of slightly, moderately, and highly competent materials. Bearing capacity analysis showed ultimate and allowable bearing capacity values high in the eastern and southern zones, while central regions exhibited reduced capacities. These results provide a good assessment for site-specific foundation design and highlight the value of using geoelectric and seismic methods in complex urban planning.

## Introduction

 To assess an engineering site, it is essential to determine the exact depth and lithology of the bedrock, locate the water table, identify lateral changes in subsurface materials, and detect any fractures, cracks, or faults. Non-invasive geophysical tools serve as cost-efficient and provide accurate results compared to conventional geotechnical tests for determining soil properties^1^.

Employing shallow seismic techniques with Electrical Resistivity Tomography (ERT) improves confidence in subsurface characterization^2^^[,3^.

**Fig. 1:**
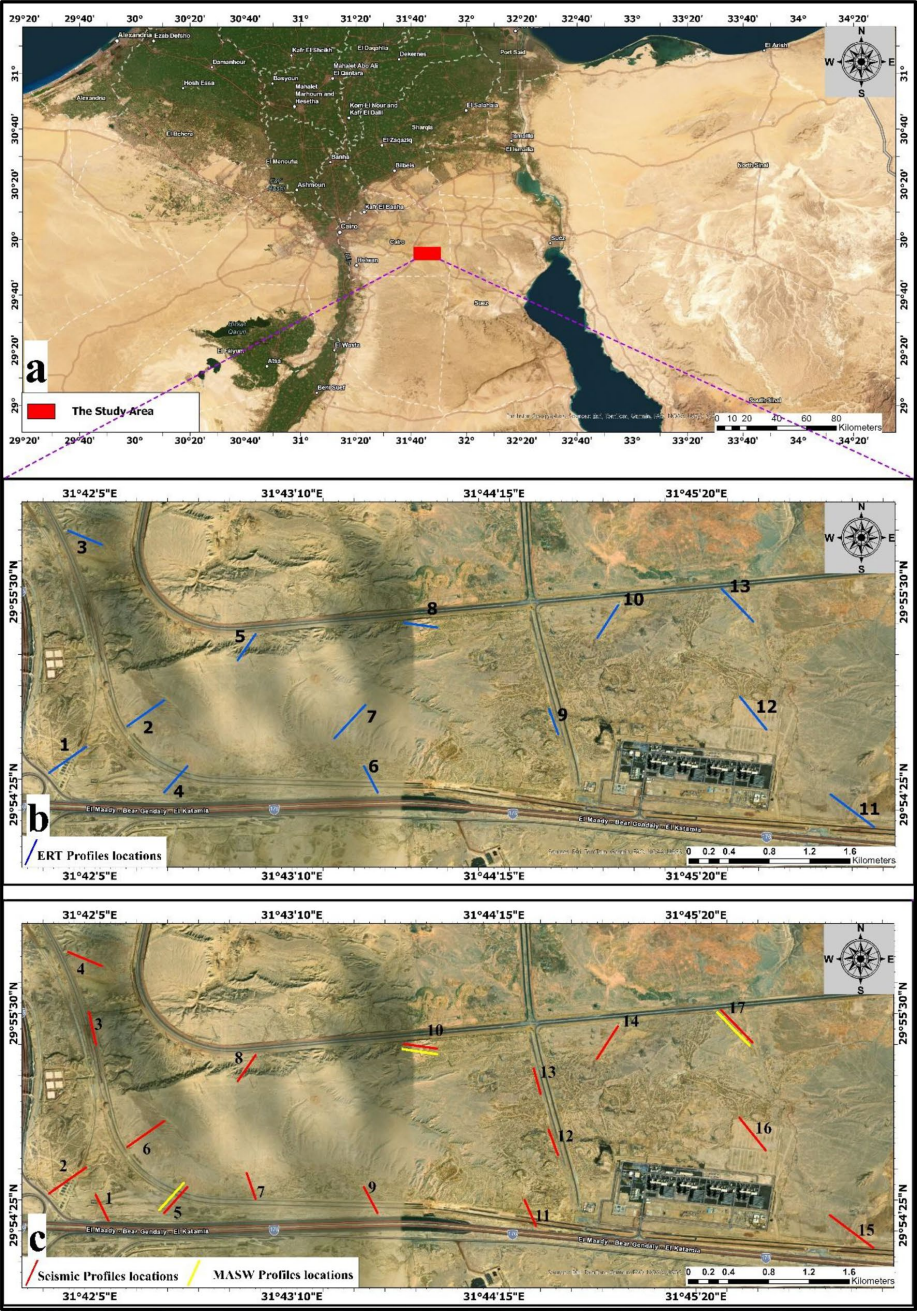
(**a**) Location map of the study area. (**b**) Location map of 2-D ERT profiles. (**c**) Location map of P wave and MASW profiles.

This study applied three distinct geophysical techniques to determine key soil parameters: Wenner geoelectric resistivity, seismic refraction, and Multichannel Analysis of Surface Waves (MASW). These approaches were used to determine specific soil parameters, including resistivity, shear-wave velocity (Vs), compressional-wave velocity (Vp), and derived elastic moduli for the subfoundation of the New Administrative Capital. The study area is located in East Cairo Fig. 1.

The ERT method is increasingly used to delineate subsurface structures and evaluate shallow geotechnical characteristics, offering high-resolution insights critical for engineering and environmental assessments^1–7^.

For shallow subsurface electrical characterization, the Wenner array configuration remains one of the most widely used resistivity techniques^8^. In this study, the Wenner method was applied to produce 2D resistivity profiles across 13 selected sites to measure the electrical resistivity of subsurface materials. This technique detects and interprets variations in soil composition, moisture content, and lithological boundaries, thereby supporting geotechnical classification. The method’s simplicity, cost-efficiency, and adaptability to different terrains make it a valuable tool for shallow site investigations. Its results were cross-referenced with seismic data to enhance interpretation accuracy and reduce ambiguity in soil classification. For shallow subsurface velocity modeling, seismic refraction is a widely used geophysical method for determining compressional-wave velocity (Vp), which reflects the speed at which longitudinal seismic waves travel through soil and rock layers^9^.

Seismic refraction was employed to determine P-wave velocities, which are critical for evaluating dynamic stiffness and estimating Moduli of elasticity, including bulk modulus and Young’s modulus. The technique involves generating seismic waves using active sources a sledgehammer, and recording their travel times across geophone arrays. The Multi-channel Analysis of Surface Waves (MASW) is a widely recognized acronym^10^^[,11^.

The shear-wave velocities in the subsurface are commonly predicted using seismic surface waves obtained from either active or passive sources. In addition to earthquakes, surface wave techniques primarily rely on active energy sources including sledgehammers, explosions, shock sources, and vibrating machines. However, modern applications obtain the surface waves from noise data using ambient noise as a passive source^12–15^^[,16^.

In this study, we applied a common, totally automated geophysical seismic method to determine the important shallow geotechnical characterization of the soil (Such as Kinetic Elastic Moduli, Material Competence Scales, and bearing capacities of shallow foundation) which yields shear wave velocity profiles crucial for subsurface investigation.

Surface wave dispersion properties are employed in this technique to obtain Vs profiles across depth (1D). one-dimensional (1D) MASW survey involves a common shot gather (CSG), where a group of receivers positioned along a (pseudo) horizontal line record the surface waves that propagate outward from the source. The 1D MASW determines the shear-wave velocity-depth profile, reflecting the average substructure underlying the geophones set^16^.

Using this method for utilization in a variety of engineering purposes has many advantages. It’s comprehensive coverage, streamlined, costless, non-invasive, relatively simple application, effectiveness and accuracy when compared with older technical and geophysical methods used in foundation investigation such as geoelectric and other methods. The Surface waves measured and evaluated through the MASW procedure are resilient and less susceptible to noise-induced contamination. This provides excellent data gathering even in difficult situations. Three MASW profiles measured in different locations. These three sites are chosen to cover every type of lithology in the research area depending on the previous geophysical interpretation data.

## Geologic setting

The study area is part of the Cairo Suez district. The deformation background and exposed geologic structures of the Cairo-Suez District (CSD) make it one of the most interesting locations in the northeastern desert for geologists 17 and 18. CSD stretches from the northern edge of the Gulf of Suez rift in the east to the Nile Valley in the west. It is a zone of substantial geological rift systems inside an unstable shelf region in northeastern Egypt^19^. CSD comprises a complicated tectonic sequence from the Mesozoic to the Cenozoic, marked by several stages of deformation involving the movements of the Eurasian, African, and Arabian plates, which can be seen from a tectonic perspective^20–22^.

The stratigraphic section in the studied region is formed of four major rock units that are organized from oldest to youngest: Wadi Hof Formation, Gebel Ahmar Formation, Gharra Formation, and Geniefa Formation^19^. The upper Eocene Wadi Hof Formation is the oldest known rock unit. It consists of interbedded limestone, claystone, and sandstone beds^23^.

**Fig. 2:**
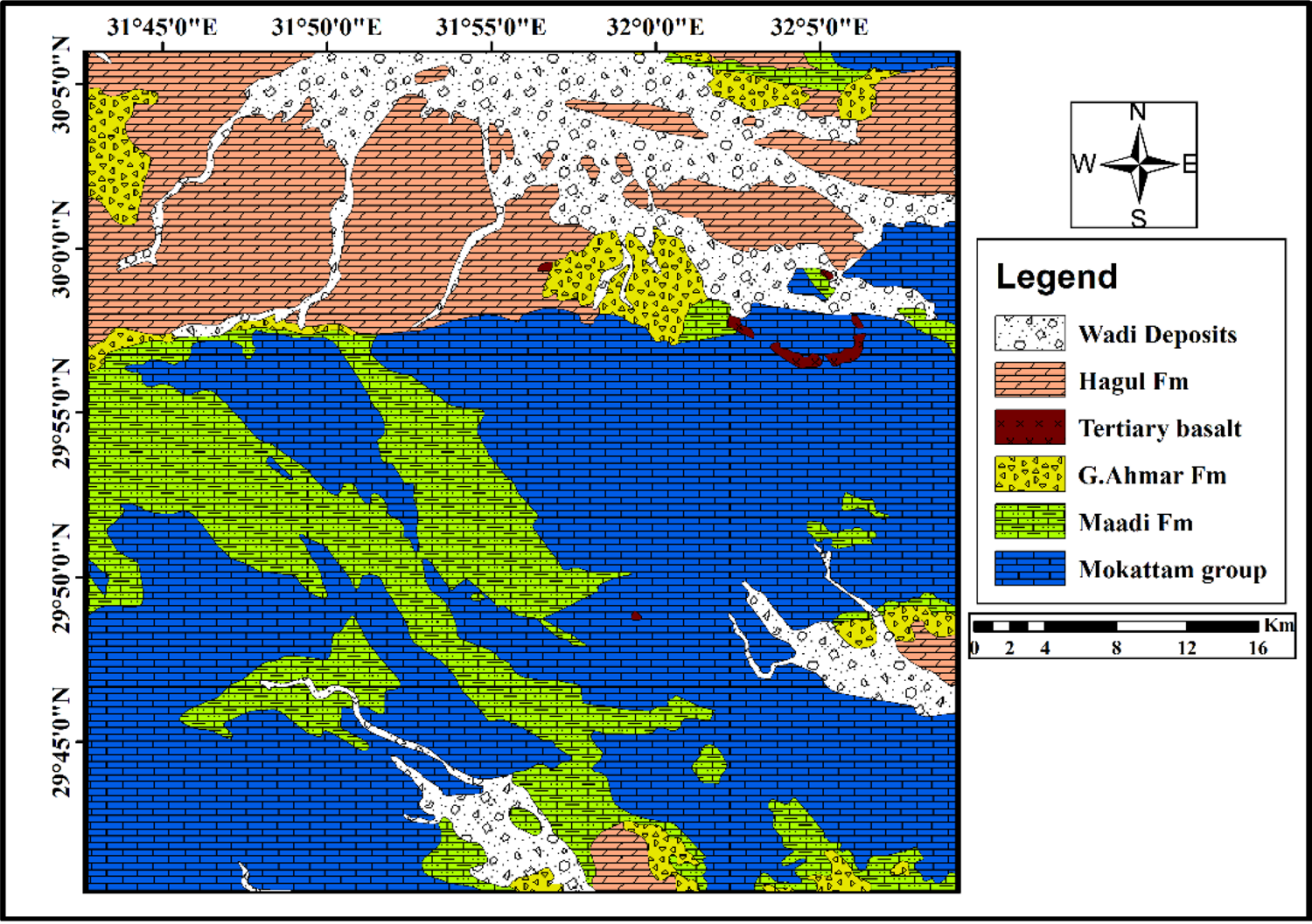
Geologic map of the study area (modified after Conco 1987).

Oligocene sediments unconformably overlie the Upper Eocene rocks and comprise the Gabal Ahmar Formation, which comprises gravels and sandstones with tree trunks and silicified pipes^24^. The late Oligocene to Early Miocene basalt flows cover the Oligocene strata^25^. The Miocene sediments, which unconformably overly the Oligo-Miocene basalt flows, are subdivided into two main formations: the Lower Miocene Gharra Formation (LMF), which is composed of sandstone, shale, and sandy limestone, and the Middle Miocene Geniefa Formation (MMF), formed of chalky limestone with marl and shale intercalations^19^. Figure 2. Show the distribution of the exposed rocks in the area under investigation.

### Surface lithologic evidence

In this study, the surface lithology observed in exposed sections across the study area reveals a diverse range of sedimentary rocks, including Sand and Gravels, sandstone, shale, and limestone. These lithological units appear in various combinations. These lithologies reflect a range of environments indicating that environmental changes have influenced the region.

### Structurally

The area under investigation is affected by normal faults directed NE-SW, NW-SE, E-W and N-S that belong to the Gulf of Suez rift opening in the late Oligocene to early Miocene^19,25,26^.

## Methodology

### Geophysical data acquisition

**Fig. 3:**
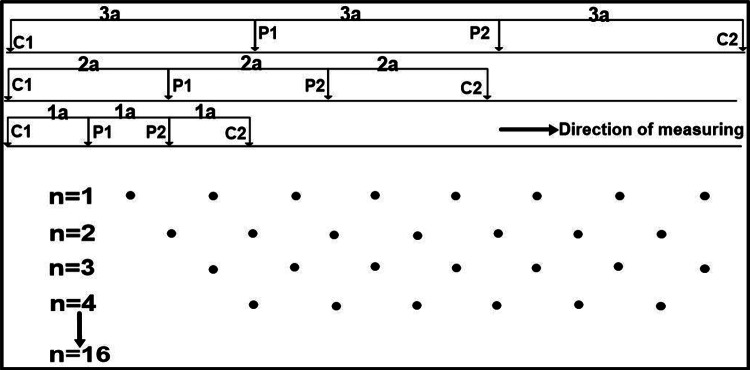
The electrode configuration for the Wenner array used for a 2D electrical survey.

**Fig. 4:**
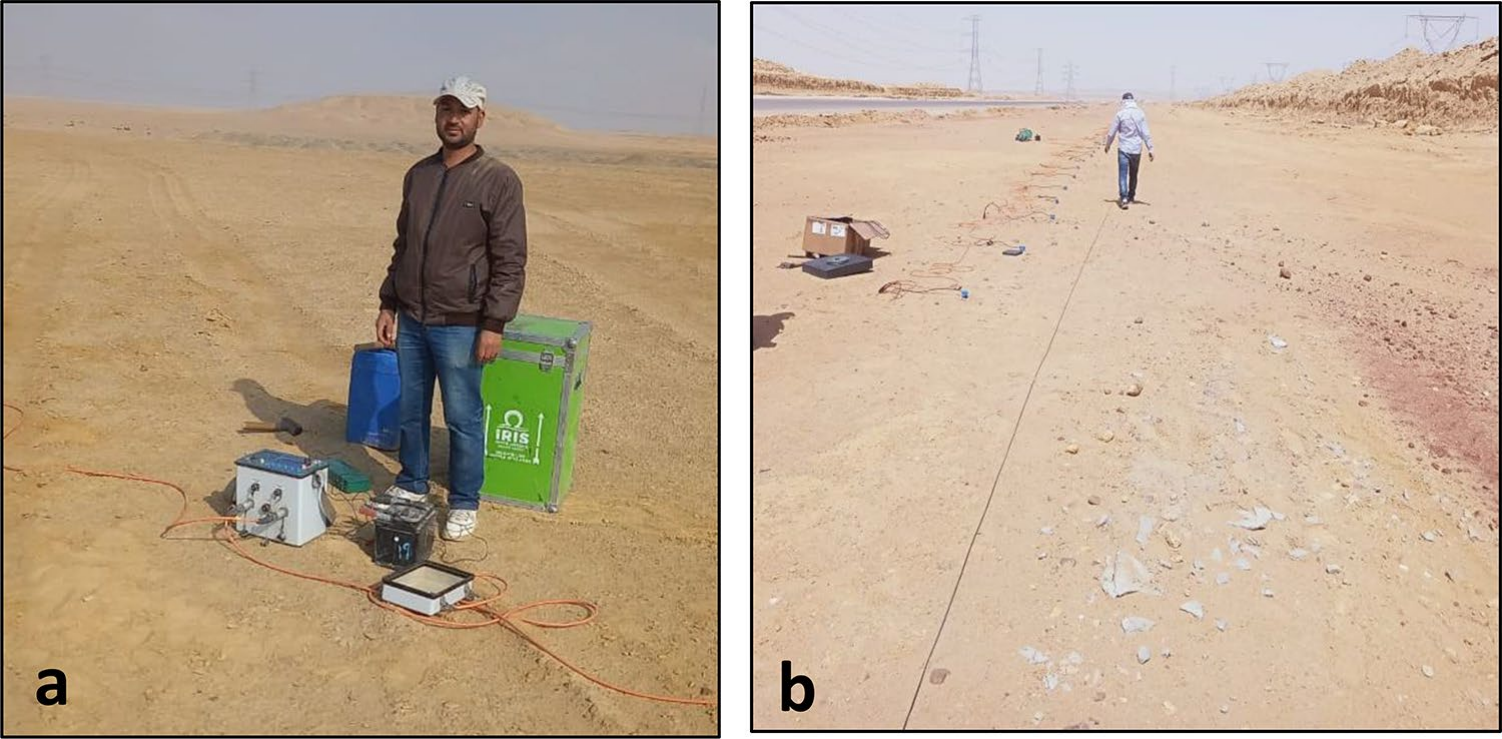
**a** Field deployment of 2D Electrical Resistivity Tomography (ERT system, illustrating the resistivity meter, and power source used for subsurface resistivity acquisition) **b** Geophones distribution of Seismic Refraction survey.

The electrical resistivity tomography (ERT) survey was utilized in this study at 13 locations (Fig. 1b) using a 2D profiling method with a Wenner array configuration. A total of 48 electrodes were placed along a straight profile with a constant spacing of 5 m (Fig. 3), resulting in a total survey length of 235 m, corresponding to an exploration depth of around 40 m. The measurements were performed using Syscal Pro Fig. 4a. In this study, the geoelectric profile is built in a single layout without applying the roll-along technique. A multi-electrode resistivity meter with automated switching was used to get apparent resistivity readings by the standard Wenner procedure, which requires symmetrical spacing between the current and potential electrodes. The investigation was principally focused on generating a 2D resistivity model for assisting in subsurface characterization and engineering evaluation.

The MASW acquisition strategy improves the lateral velocity resolution and provides reliable results^27^. A preliminary test profile was carried out at the site to identify the ideal acquisition settings, which included adjusting the suitable source type, the suitable geophone positions and spacing, and the suitable offset (Distance from shot to first geophone in meters = array distance).

**Fig. 5:**
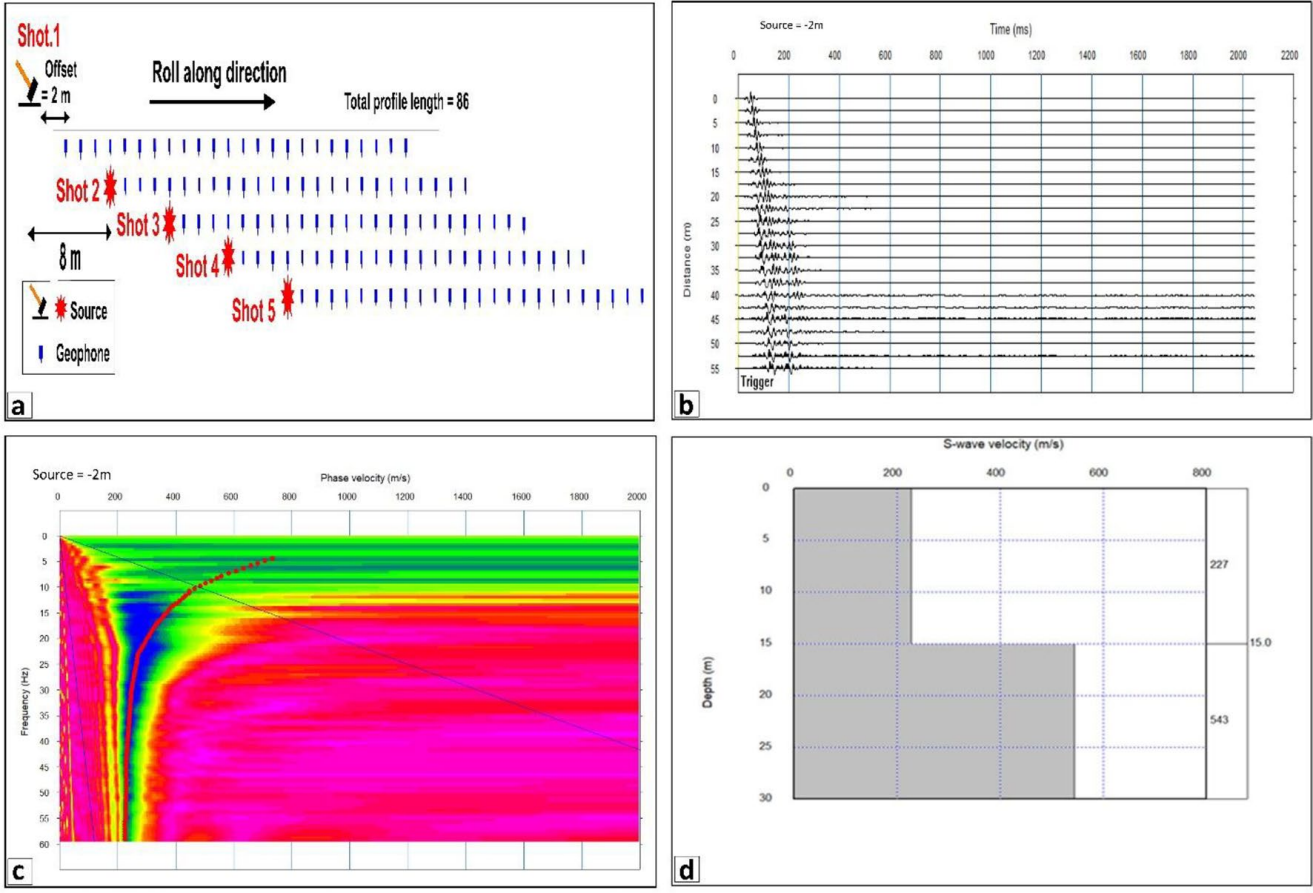
**a **Schematic representation of MASW spread method **b**. multichannel record c dispersion curve d 1D shear wave profile.

Depending on the initial experiment to create accurate data, MASW data were gathered and recorded using 24 Geophones (receivers) with 4.5 Hz frequency and spacing 2 m between geophones, by using sledgehammer (weight = 10 Kg) as a source of energy to create waves and to guarantee improved contact between sledgehammer and the ground and enhance wave propagation’s efficiency a metallic plate was utilized. The offset (The separation between the initial geophone and the source) was 2.5 m, making the total spread length 57.5 m. The measurements were reanalyzed by modifying the field arrangement (shifting 10 m) and analyzing five sets of ground roll to image shallow surface heterogeneity. The schematic representation of the MASW spread method is shown in Fig. 5a.

To determine the subsurface stratigraphy, a seismic refraction study was conducted. The seismic configuration utilizes by using 24 vertical geophones with 14 Hz frequency, providing sensitivity to low-frequency P-wave energy ideal for engineering-scale investigations. The Geophone spacing (Geophone interval) was 5-meter along the profile, Fig. 4b. A sledgehammer was used to generate P-wave energy, which was recorded by a multichannel seismograph with accurate synchronization of the trigger. Five shot points were executed to optimize ray coverage and enhance velocity model resolution. The first shot was placed 2.5 m ahead of the first geophone, while the second and fourth shots were placed in the interstitial spaces between geophones 6–7 and 18–19, respectively. The third shot was positioned in the middle of the profile to guarantee symmetrical wave propagation, while the fifth shot was positioned 2.5 m apart from the final geophone to extend the ray path coverage. The shot points spacing was 10 m to provide sufficient horizontal resolution and depth penetration. The acquisition geometry was optimized to ensure a good subsurface coverage and minimize blind zones. The distribution of P waves and seismic profiles shown in Fig. 1c.

### Geophysical data processing and inversion

The setup for geoelectric processing of 2D resistivity profiles requires an organized workflow that converts raw field information into interpretable subsurface models. The method begins with data acquisition, which involves recording apparent resistivity values using resistivity meter and electrodes. Following data collection, field results are arranged into a formatted input file suitable for inversion analysis. RES2DINV is a commonly used application for the next step, which builds a true resistivity image of the subsurface using 2D inversion of apparent resistivity datasets. The program iteratively modifies the model using a finite-element or finite-difference algorithm until the calculated apparent resistivities match well with the measured data, maintaining an appropriate error level. Before inversion, it is necessary to verify electrode geometry and remove noisy data points as a preprocessing step. Finally, the post-processing steps involve assessing the resistivity profiles in order to detect zones with varying resistivity that may reflect various lithologies, degrees of moisture, or structural characteristics.

MASW data processing involves a sequence of steps designed to extract subsurface shear wave velocity (Vs) profiles. The first step of the procedure is CMP (Common Midpoint) gathering, in which field layout data is entered manually after data format conversion. Cross -correlograms were created by performing the Fast Fourier Transform (FFT) to shot gather waveforms (example shown in Fig. 5b) and deriving cross spectra for each pair of channels. The data is then processed by velocity analysis to produce a set of phase velocities as a function of frequency (Fig. 5c) by utilizing highest possible amplitude of stacked CMP gathers at each frequency. These curves are then inverted to produce 1D Vs profiles (Fig. 5d) for shallow subsurface layers. Finally, multiple 1D profiles are combined to construct a comprehensive 2D Vs velocity structure, offering a detailed view of subsurface conditions.

**Fig. 6:**
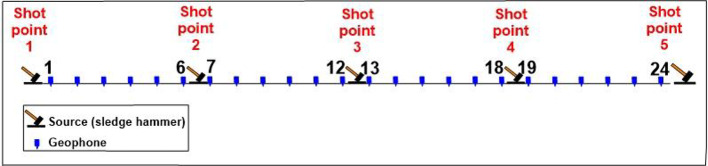
Geophone spread for a refraction survey with shot locations indicated.

**Fig. 7:**
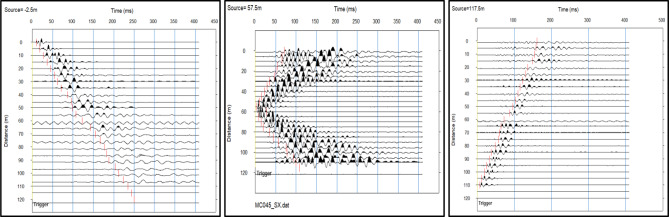
Seismic waveforms recorded from three shot points (SP −2.5 m, SP 57.5 m, SP 117.5 m), showing first arrival times across geophone positions.

**Fig. 8:**
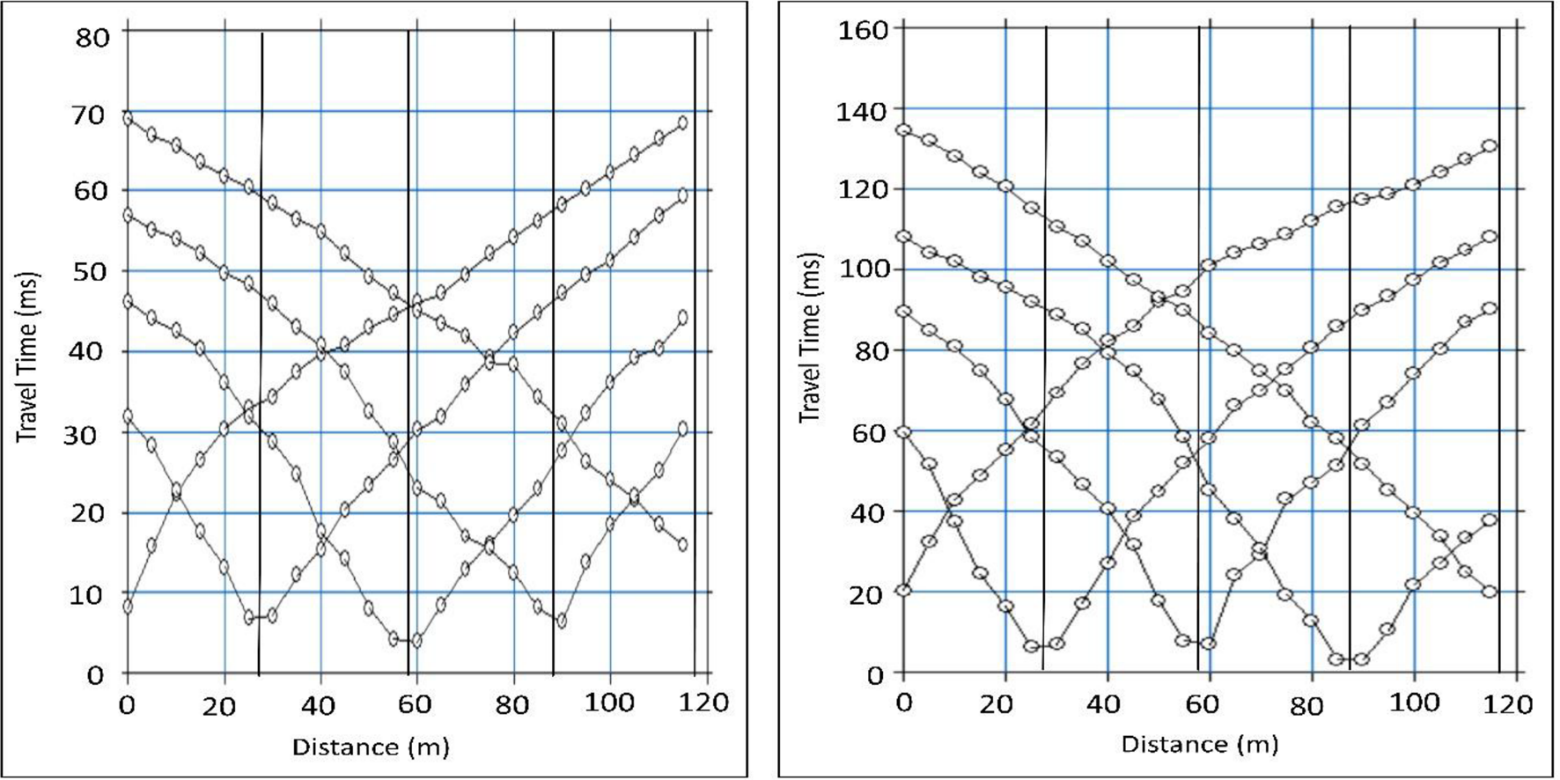
interpreted travel-time distance curve.

Compressional wave (P-wave) processing involves an organized procedure to identify subsurface layers and determine the material’s parameters. After collecting seismic data using geophones and a surface source in the acquisition step (Fig. 6), the processing step recognizes the first arrival of P-waves across multiple shot recordings (example shot gathers shown in Fig. 7). The recorded waveforms are imported into SeisImager, which is used to visualize the data and manually pick the first breaks. These picks are then exported and input into ZondST2 to perform inversion to build subsurface velocity models. Picked travel times are plotted as traveltime versus distance (Fig. 8) to identify linear segments and apparent velocities before inversion. Generating 2D velocity sections is the final step that reveals layer boundaries, material types, and geotechnical characteristics required for site assessment and foundation planning.

**Fig. 9:**
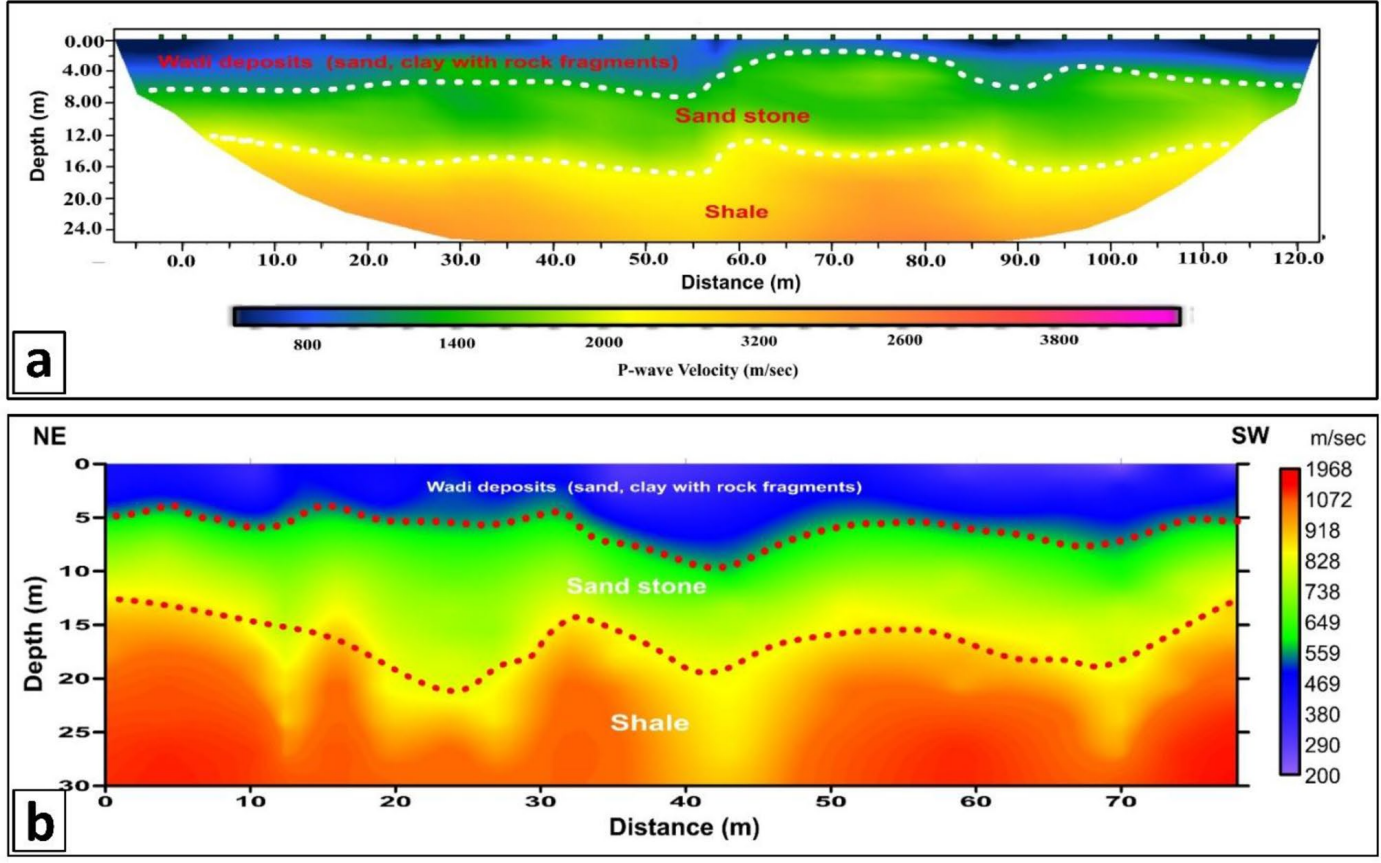
2D shallow seismic velocity models. (**a**) P-wave velocity (Vp) profile (seismic refraction tomography) and (**b**) S-wave velocity (Vs) profile (MASW inversion). Both models clearly show the interpreted lithological units: Wadi deposits, Sandstone, and Shale. Note the high velocities observed in the Shale layer, indicative of its highly compacted and competent nature. Dashed lines mark unit boundaries.

The final output of this comprehensive seismic processing workflow is visualized in Fig. 9, which represents the complete 2D P-wave velocity field and its companion S-wave velocity model, highlighting the significant contrast in velocities between the unconsolidated deposits and the deeper Shale, where high velocities are observed due to its highly compacted and competent nature in the study area.

### Derivation of geotechnical parameters from seismic velocities

Laboratory techniques for determining geotechnical parameters are accurate but are often expensive and spatially constrained. Conversely, geotechnical investigation employing seismic velocities provides comprehensive, non-invasive, time-saving, and cost-efficient data, making it commonly used for engineering purposes.

Seismic velocity data collected from 17 P-wave refraction sites and 3 MASW locations enabled the calculation of key geotechnical parameters across the study area. The MASW sites were carefully selected based on geoelectric profiles that captured all major lithologic types, ensuring that shear wave velocity (Vs) measurements represented the area’s subsurface variability. The derivation of these parameters is presented in the following sections. Among the most important elastic parameters derived from seismic velocities are Poisson’s ratio, rigidity modulus, Young’s modulus, and bulk modulus, which collectively describe the soil’s elastic response to applied stresses.

Characterization of soil stress–strain behavior is a critical component of geotechnical design, informing applications such as site characterization, settlement analysis, seismic hazard assessment, site response evaluation, and soil–structure interaction 30 and 31. Among the most important elastic parameters derived from seismic velocities are Poisson’s ratio, rigidity modulus, Young’s modulus, and bulk modulus, which collectively describe the soil’s elastic response to applied stresses.

Poisson’s ratio (σ): The parameter quantifying the relationship between the shape change parallel to the applied stress and the shape change perpendicular to the applied stress as a negative ratio (negative transverse to axial strain ratio) is defined as Poisson’s ratio (σ)^32^. It is close to zero for extremely hard indurate rocks, half for liquids and turns negative for highly anisotropic hard rocks^33^. In theory, partially saturated clays have σ = 0.3–0.4 and saturated clays have σ = 0.5. The standard σ measurements of sands and silts range from 0.4 in a dense condition to 0.2 in a loose state^34^.

The following equation is employed to estimate the Poisson’s ratio $\:{\upsigma\:}$^35^.

1 $$\:{\upsigma\:}=\frac{\varvec{\lambda\:}}{\left[2\left(\varvec{\lambda\:}+\varvec{\mu\:}\right)\right]}$$

λ and µ represent the first and second Lamé constants respectively, defined by λ = ρ⋅(Vp2 − 2Vs2) and µ = ρ⋅Vs2. The equation is reformulated to:

2 $$\:{\upsigma\:}=\frac{[{\left(\frac{\varvec{V}\varvec{p}}{\varvec{V}\varvec{s}}\right)}^{2}-2]}{\left[2\:\right({\left(\:\frac{\varvec{V}\varvec{p}}{\varvec{V}\varvec{s}}\right)}^{2}-1\left)\right]}$$

Where σ is Poisson’s ratio (dimensionless), Vp and Vs represent the velocities of P-waves and S-waves respectively, measured in meters per second, m/s.

Once Poisson’s ratio was determined, it was used together with Vs and density to calculate other elastic moduli that describe the deformation characteristics of the soil.

The rigidity modulus (µ), also known as shear modulus, is a crucial dynamic characteristic in geotechnical engineering. It describes the soil’s resistance to distortion induced by shear wave propagation and is calculated from the formula below according to elastic theory^36^^37^,

:3 $$\mu \: = \rho \:\:{\text{v}}_{\text{s}}^2\:$$

Where = shear modulus (in Kpa- psf); Vs = phase velocity of the S-wave (in m/s or ft/sec); and ρ is density (in kg/m3 - gm/cm3).

To evaluate the material’s response under axial loading, Young’s modulus (E) was calculated. It describes the resistance of soil to elongation or compression when forces are applied is described by Young’s Modulus (E).

The formula below can be used to compute the modulus of elasticity (E) in terms of Poisson’s ratio and shear modulus^38^.:

4 $${\text{E = 2}}\mu {\text{(1 + }}\sigma {\text{)}}$$

Where µ represents the shear modulus and σ denotes Poisson’s ratio.

Alternatively, E can be derived directly from Vp, Vs, and density (ρ) according to the following relationship^39^:

5 $$\:\varvec{E}=\frac{\varvec{\rho\:}{\varvec{V}}_{\varvec{s}}^{2}\left(4{\varvec{V}}_{\varvec{p}}^{2}-3{\varvec{V}}_{\varvec{s}}^{2}\right)}{{\varvec{V}}_{\varvec{p}}^{2}-{\varvec{V}}_{\varvec{s}}^{2}}$$

The bulk modulus (K) was also determined to measure the material’s ability to change in volume per unit change in applied pressure. In seismic studies, bulk modulus aids in understanding pressure wave propagation through materials and is calculated depending on shear modulus, Poisson’s ratio and Young’s Modulus from the equations below^40^.:

6 $$K = \frac{E}{{3\left( {1 - 2\sigma } \right)}}\frac{{2\mu \left( {1\: + \:\sigma \:} \right)}}{{3\left( {1 - 2\sigma \:} \right)}}$$

The material index (Mi) serves as a numerical indicator of a material’s compactness and ability to resist distortion that depends on several characteristics, such as the material’s substance, liquid in voids, fracture existence, degree of consolidation and the conditions of the environment^32^.

The material index (Mi) formulation incorporates Poisson’s Ratio (σ) as follows

7 $$\:{\varvec{M}}_{\varvec{i}}=\left(1-4\:\varvec{\sigma\:}\right)$$

In addition to Mi, the concentration index provides further insight into the material’s compactness and stress distribution. For civil and geotechnical applications such as foundation design, the concentration index quantifies the degree of compaction and stress concentration in soils and rocks^33,41^. The concentration index is obtained using Vs and Vp through the following expression^42^:

8 $$\:{\mathbf{Ci}}{\text{ }} = {\text{ }}\left( {{\mathbf{3}}{\text{ }} - {\text{ }}{\mathbf{4\alpha }}} \right){\text{ }}\left( {{\mathbf{1}} - {\mathbf{2}}{\text{ }}{\mathbf{\alpha }}} \right)$$

Where α = (Vs^2^/Vp^2^).

To further characterize subsurface stress conditions, the stress ratio (Si) was computed. In geotechnical studies, Stress Ratio (Si) is a dimensionless coefficient to evaluate the interaction between a soil’s effective stress and pore pressure. Geological formations with high fluid pressure tend to have reduced differential pressure and remarkably low seismic wave propagation velocities. This is because the reduction in effective stress caused by high fluid pressure within the formation ultimately decreases the soil’s shear strength.

Based on Poisson’s ratio, the stress ratio can be calculated as follows^32^:

9 $${\mathbf{Si}}{\text{ }} = \frac{\sigma }{{1 - 2\sigma }}$$

Finally, the density gradient (Di) was estimated to assess changes in soil density with respect to stress variations. According to^43^, the density gradient can be expressed as a function of VP and σ.

10 $${{\mathbf{D}}_1}{\text{ }} = {\text{ }}\left( {{\mathbf{3}}{\text{ }}/{\text{ }}{{\mathbf{V}}_\rho }} \right){\text{ }} - {\text{ }}(\left( {{\mathbf{1}}{\text{ }} - {\text{ }}{\mathbf{\sigma }}} \right){\text{ }}/{\text{ }}({\mathbf{1}}{\text{ }} +$$

High density gradient values of soil often reflect its softness, as small pressure variations can significantly reduce porosity and increase density. Conversely, hardened rock tends to produce lower gradient values^44^.

#### Foundation bearing capacities

a. Ultimate bearing capacity (Qult).

It is the highest load at which the foundation transitions into shear failure and is directly dependent on its shear strength. Researchers have widely proposed empirical relationships for determining soil bearing capacity^45–49^.

Qult (in kg/cm²) is calculated based on bulk density (ρ, in gm/cc) and Vs (in m/s), as outlined below:

11 $${{\mathbf{Q}}_{{\mathbf{ult}}}} = {\text{ }}\left( {{\mathbf{\rho }}*{\mathbf{VS}}} \right)/{\mathbf{100}}$$

A relationship was formulated by^50^ to calculate Qult using Vs, expressed by the following equation.

12 $$Log Qult =2.932 (logVS - 1.45)$$

Many researchers predict Qult depending on Vp^51^. redict equation between Qult and Vp as shown below:

13 $$\:\mathbf{Q}\mathbf{u}\mathbf{l}\mathbf{t}=0.0005\:{\varvec{V}}_{\varvec{p}}^{1.1042}$$

b. Allowable bearing capacity (Qall).

Qall is derived from the ratio of Qult to the safety factor (F), as expressed in the following formula:

14 $$Qall = Qult/F$$

In this investigation, we utilized a safety factor set at 3^51^. Predict a formula between Qall and Vp as the follows:

15 $${Q_a} = 0.0001V_p^{1.1028}$$

## Results and discussions

### Electric resistivity tomography

The geoelectric survey comprises thirteen subsurface profiles from P1 through P13. The 13 geoelectric profiles exhibit distinct subsurface properties, soil composition variations, and structural formations, impacting geotechnical stability and foundation feasibility. These profiles reach investigation depths of up to 40 m and extend horizontal distances of 235 m, except for P2 and P3 (115 m length) and P6 (175 m length). The directional orientations are as follows: P1 (SW–NE), P2 (NE–SW), P3 (SE–NW), P4 (NE–SW), P5 (NE–SW), P6 (NNE-SSW), P7 (SW-NE), P8 (W-E), P9 (N-S), P10 (NE-SW), P11 (NW-SE), P12 (NW-SE), and P13 (NW-SE). The profiles display resistivity values ranging from less than 1 Ohm·m to over 1000 Ohm·m, visualized through a color gradient from dark purple (low resistivity) to dark red (high resistivity). These variations help distinguish between conductive materials such as saturated clay and more resistive units like sandstone or fractured limestone. Surface geological exposures were documented to support and strengthen geophysical interpretations. By correlating observed lithologies and structural features with subsurface anomalies, the study reinforces the reliability of the stratigraphic framework and enhances the accuracy of soil classification and structural analysis.

**Fig. 10:**
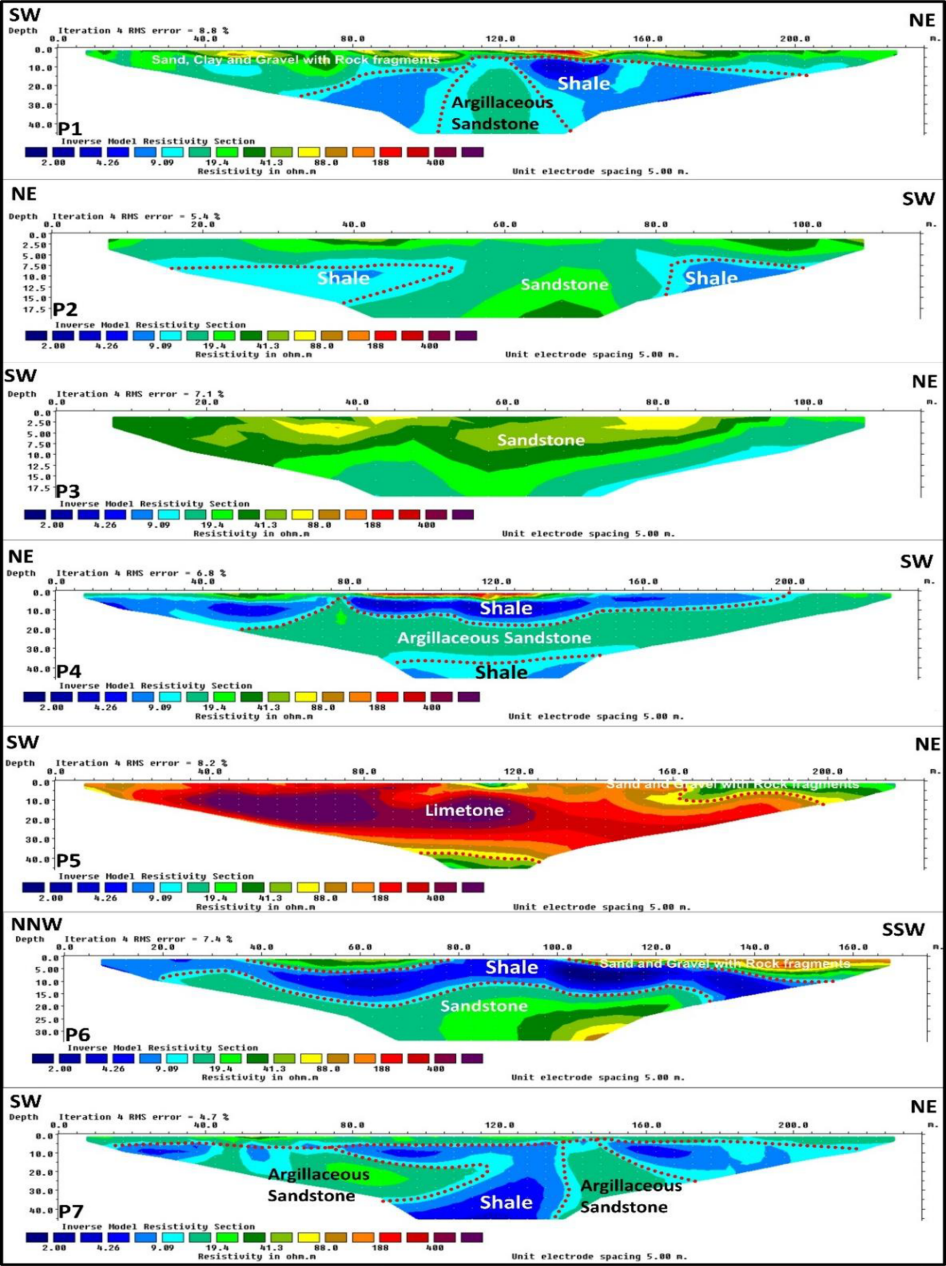
Electrical Resistivity Tomography profiles 1, 2, 3, 4, 5, 6, and 7.

**Fig. 11:**
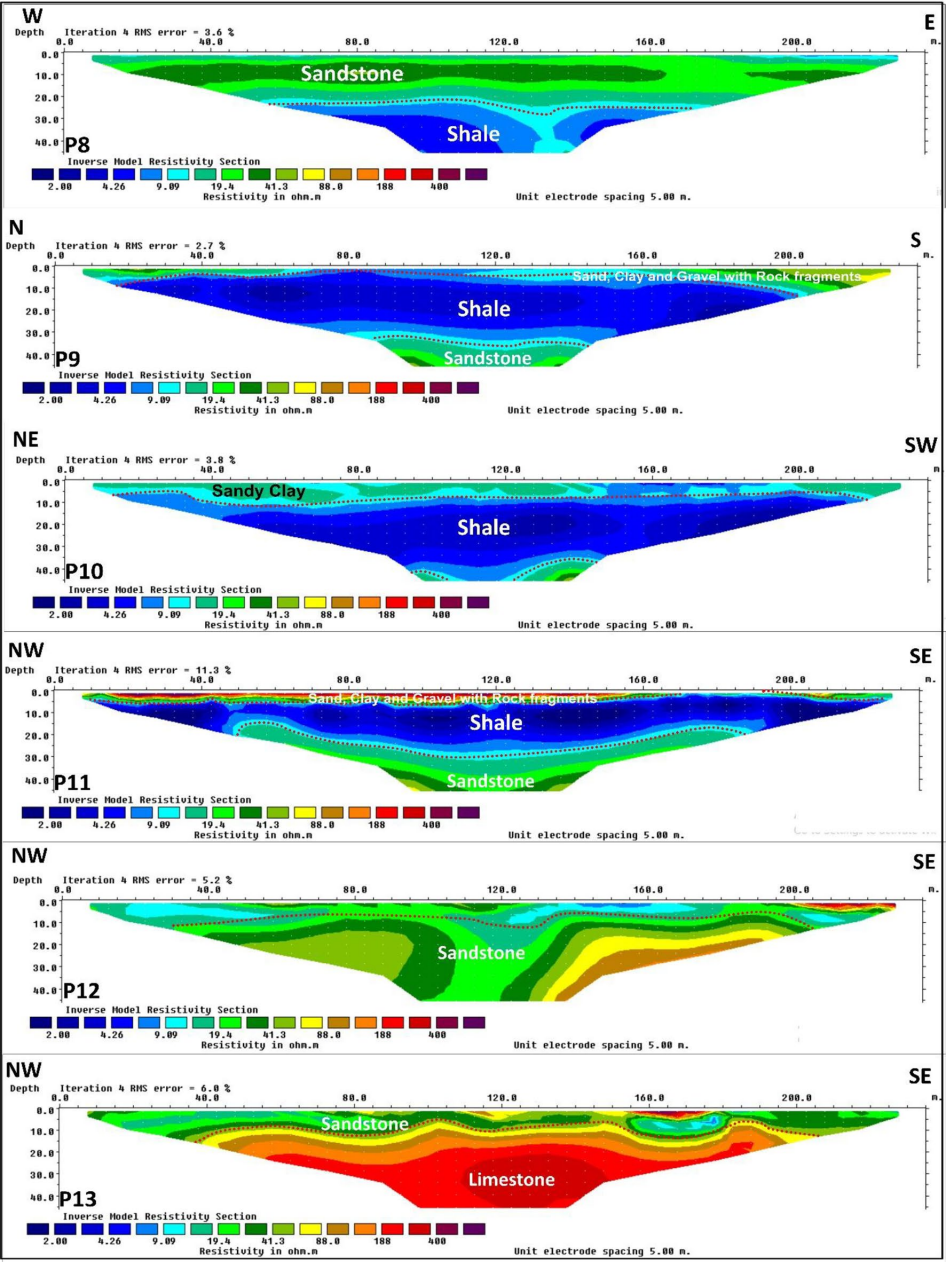
Electrical Resistivity Tomography profiles 8, 9, 10, 11, 12, and 13.

**Fig. 12:**
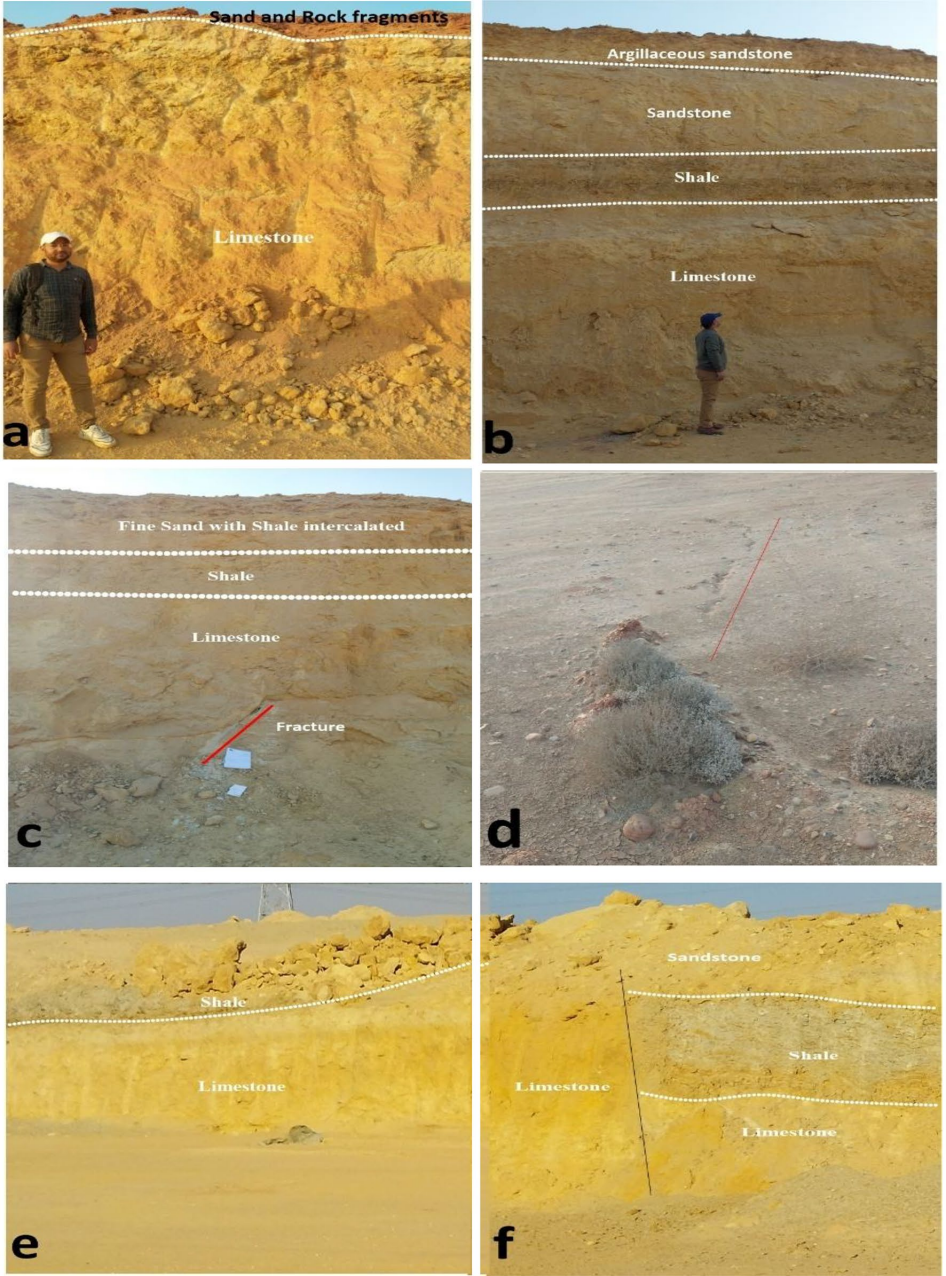
Field photographs (**a**–**f**) showing surface exposures of sand, sandstone, shale, and limestone and dominant structural features such as fractures.

The 13 ERT profiles (Figs. 10 and 11) reveal three subsurface layers with distinct lateral variations. The first layer, observed in profiles 1, 2, 4, 5, 6, 7, 9, 11, and 13, is thin and characterized by varied resistivity values related to sand, clay, and rock fragments. Profiles 10 and 12 show notably low resistivity in this layer, suggesting higher clay content. This layer exhibits a thin thickness and is visibly exposed in surface outcrops shown in Figure (12 a). This layer is absent in profile 3, which reflects a thick sandstone layer as the first unit that extends vertically from the surface to the end of the investigation. The second layer: Noticeable lateral changes characterize the second subsurface layer throughout the surveyed area. This layer indicates very low resistivity values ranging from 2 to 10 Ohm·m, exhibiting a shale layer with thicknesses varying from 8 m to about 30 m. This layer appears as a continuous unit at profiles 3, 4, 6, 8, 9, 10, and 11. As illustrated in Figure (12 b and c), the shale layer also appears prominently in surface exposures, confirming its lateral continuity and supporting the geophysical interpretation. In contrast to the continuous shale layer observed in several profiles, profiles 1, 2, and 7 reveal a significant discontinuity where resistivity values increase laterally (ranging from 15 to 100 Ohm·m), indicating a lateral transition to sandstone. These sandstone resistivity range depends on cementation, porosity, and saturation degree.

Profiles 5 and 13 are distinguished by a limestone unit as a second layer, characterized by high resistivity values ranges from 100 to 600 ohm·m. with a thickness of about 30 m and continued to the end of the investigation at profile 13. Surface exposures shown in Figure (12 a, b, c and f) also reveal extensive limestone outcrops, supporting the geophysical interpretation of this layer. Several surface exposures reveal prominent fracture zones in the study area, particularly within the limestone units Figure (12 c and d).

As shown in Figure (12. f), surface exposures also reflect this lateral lithologic variability in different localities along the study area. These field observations support the geophysical interpretation of lateral facies changes and reinforce the stratigraphic complexity across the study area.

The third layer is identified in profiles 3, 5, 6, 9, 10, and 11. This deeper unit represents a consolidated lithology beneath the shale or limestone layers and consists of sandstone with resistivity ranging from 15 ohm·m to 100 ohm·m.

### Geotechnical parameters from shallow seismic measurements

Before presenting the geotechnical results, it is essential to outline the general subsurface conditions of the study area, as the lithologic variations strongly influence the elastic, mechanical, and bearing properties of the materials. The integrated interpretation of the seismic velocity and electrical resistivity profiles reveals two main subsurface layers with variable composition and thickness. The upper layer generally consists of unconsolidated to semi-consolidated sand and clay with rock fragments, while the lower layer comprises more competent materials such as sandstone, shale, and fractured limestone. A simplified lithologic summary of the first and second layers along the investigated profiles is presented in Table 1.

**Table 1:** Simplified lithologic summary of the first and second subsurface layers interpreted from geophysical and geotechnical data across the study profiles.

Profile no.	First layer lithology	First layer thickness (m)	Second layer lithology	Second layer thickness (m)
1, 2, 3, 5, 6, 10, 11, 13, 16	Sand and clay with rock fragments	4–14	Sandstone	8–24
4, 12, 14, 15	Sandy clay/Sandstone	2–12	Shale	6–28
8, 17	Sand and clay with rock fragments	2–12	Fractured limestone	8–20
9	Weathered shale	6–12	Sandstone	6–12
7	Highly Weathered Fractured limestone	6–8	Fractured limestone	16–18

**Fig. 13:**
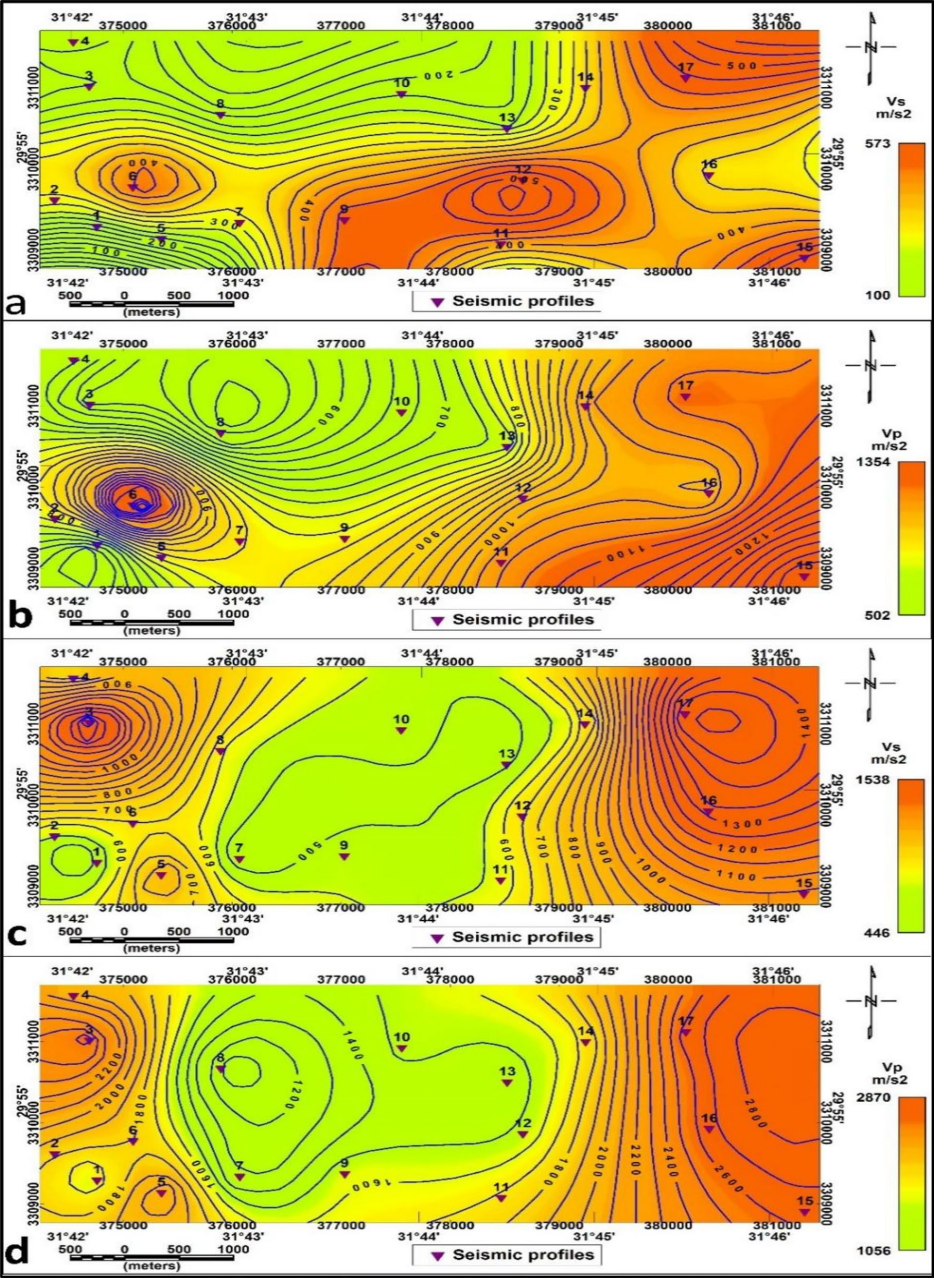
Contour maps showing seismic velocity distributions across the study area: (**a**) Shear wave velocity (Vs) in the surface layer, (**b**) Compressional wave velocity (Vp) in the surface layer, (**c**) Shear wave velocity (Vs) in the second layer, (**d**) Compressional wave velocity (Vp) in the second layer.

The contour maps in Fig. 13a-d illustrate the P-wave (Vp) and S-wave (Vs) velocity distributions across the first (surface) and second (deeper) stratigraphic layers, offering insights into subsurface material properties. Specifically, Fig. 13a and b show the Vs and Vp distributions for the first layer, respectively, while Fig. 13c and d show the Vs and Vp distributions for the second layer. Lower velocities dominate the central and northeastern zones in the surface layer, suggesting unconsolidated or saturated sediments, while higher velocities in the southwest indicate more compact lithologies. The second layer exhibits generally elevated velocities with smoother gradients, consistent with deeper, more consolidated formations. The rock and soil characteristics such as elastic strength and density are directly correlated with seismic wave velocities (shear and compressional waves) and offer a good basis for identifying geotechnical parameters^27,28^, and^29^. Using P-wave and S-wave velocities, rigidity modulus, bulk modulus, Young’s modulus, and Poisson’s ratio were computed in this study, revealing significant spatial variation in material stiffness and competence. The geotechnical parameters for the surface and bedrock layer were estimated (Table 2), respectively.

**Table 2:** Geotechnical parameters ranges in the study area.

Layer No.		Vp (m/s)	Vs (m/s)	σ	µ (MPa)	E (MPa)	K (MPa)	Ci	Mi	Si	Q_ult_ (Kg/Cm^2^)		Q_all_ (Kg/Cm^2^)	Di
First Layer	**Max.**	1373	570	0.458	564	1409	2978	5.028	0.007	0.846		6.743	2.25	−0.368
	**Min.**	500	174	0.248	44	127	307	3.182	−0.833	0.330		0.208	0.07	−0.599
	**Aver.**	877	333	0.403	222	605	1190	3.587	−0.612	0.694		2.042	0.68	−0.419
Second Layer	**Max.**	2705	1557	0.456	5407	13,462	12,314	5.082	0.020	0.837		128.348	42.78	−0.374
	**Min.**	1000	413	0.245	311	892	967	3.195	−0.822	0.324		2.622	0.87	−0.606
	**Aver.**	1860	784	0.393	1544	4134	6047	3.653	−0.573	0.667		28.047	9.35	−0.441

where, Vp = P-wave velocity (m/sec), Vs = S-wave velocity (m/sec), σ = Poisson’s ratio, Ci = concentration index, Mi = material index, Si = Stress ratio, Qult = ultimate bearing capacity (Kg/Cm^2^), Qall = allowable bearing capacity (Kg/Cm^2^), µ = Shear modulus (MPa), E = young’s modulus (MPa), K = Bulk modulus (MPa), and, Di = density gradient.

The soil characterization can be described using these parameters in terms of mechanical and geotechnical features.

#### Kinetic elastic moduli

The distribution of elastic parameters across the study area is illustrated through Poisson’s ratio, rigidity modulus, Young’s modulus, and bulk modulus maps, which together provide an integrated picture of subsurface stiffness and material response.

**Fig. 14:**
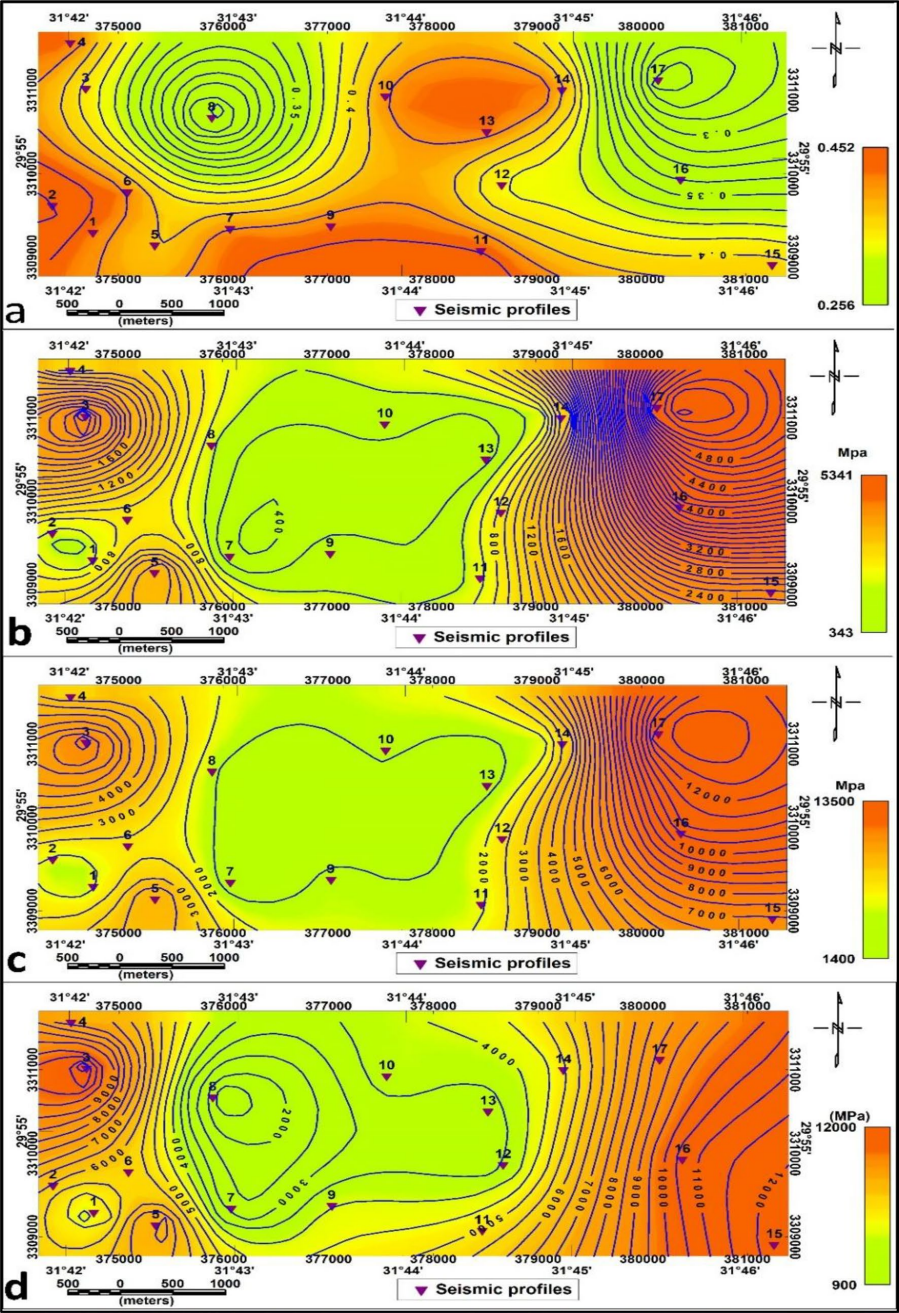
Elastic Moduli distribution maps of the first layer for (**a**) Poisson’s ratio (*σ*) (**b**) Rigidity modulus (*µ*), (**c**) Young’s modulus (**E**) and (**d**) Bulk modulus (K).

**Fig. 15:**
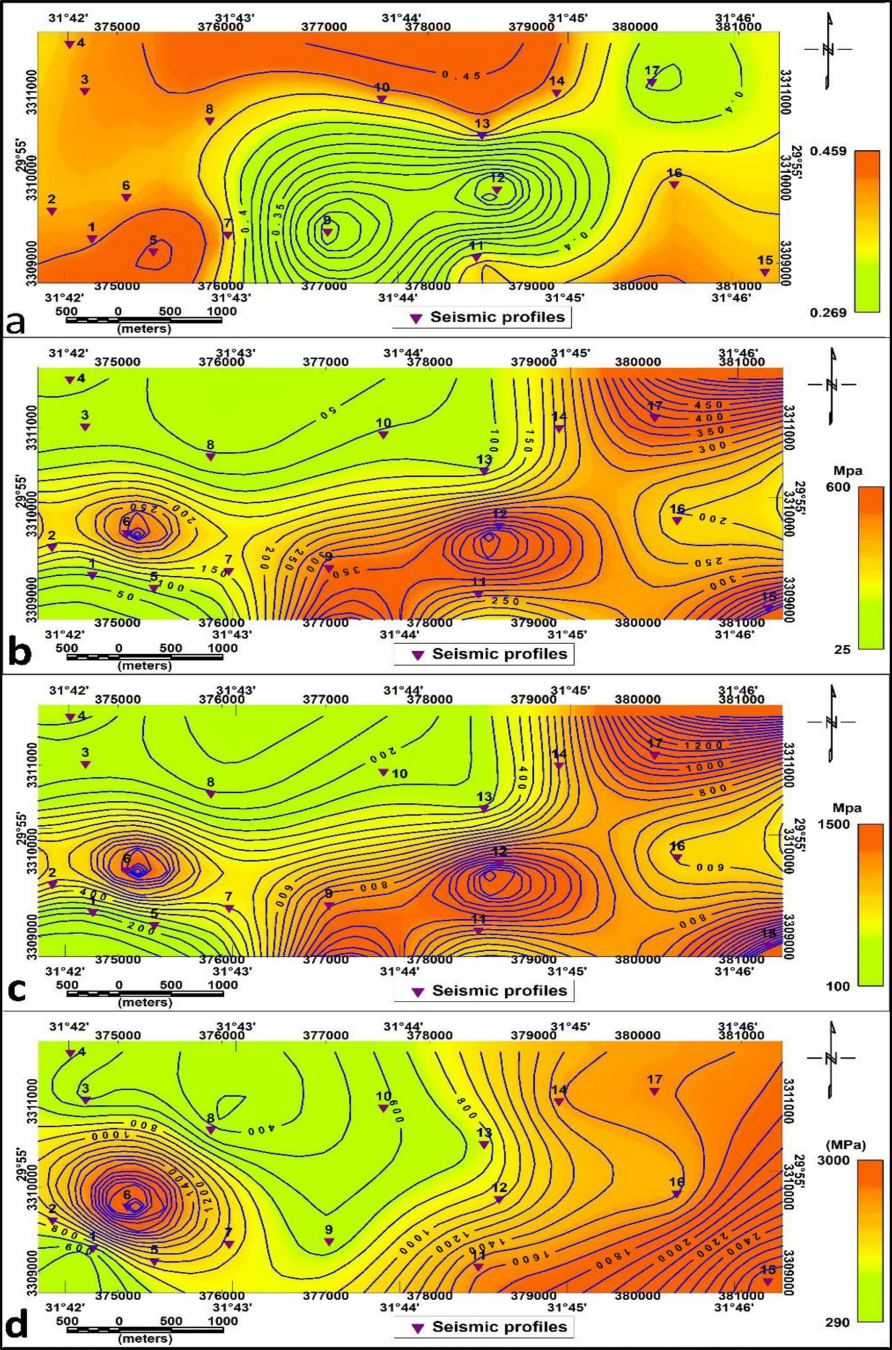
Elastic Moduli distribution maps of the second layer for (**a**) Poisson’s ratio (*σ*) (**b**) Rigidity modulus (*µ*), (**c**) Young’s modulus (E) and (**d**) Bulk modulus (K).

The Poisson’s ratio (σ) values (Fig. 14. a) are high in the northern and western parts of the study area and low values in the central parts. The second layer is characterized by low values in the northeastern part and at p8, and high values in the central part (Fig. 15.a.) indicating variation in lateral compressibility between the layers.

The Rigidity Modulus (µ) contour map (Fig. 14. b) shows values ranging from 44 MPa to 564 MPa, with the highest values localized in the eastern. The southwestern and northern parts are identified as less rigid and softer rocks. The low shear modulus values in the study area are related to less rigid and softer rocks and high values for fairy competent material.

The values of this modulus change to range from 311 to 5407 in the second layer, where the high values shown in both the eastern and western sides reflect high competent material, which decreases low values in the central parts related to fairy competent soil (Fig. 15. b).

The Young’s modulus (E) contour map (Fig. 14. c.) illustrates a wide variety of readings from about 127 MPa to 1409 MPa, indicating a substantial variance in the material’s stiffness beneath the surface of the study area. Young’s modulus map reveals slightly and fairly to moderate competent materials in the area under study. On the other hand, the second layer has a ranges of 1452–13,462 MPa. The high values in the northern and southern parts of the research area signify highly competent and the central portion is fairy competent, characterized by low values (Fig. 15. c.). Based on typical categories, the Young’s modulus () map’s results are in excellent compliance with acknowledged geomechanical defining features. Sedimentary rock typically ranges between 100 and 500 MPa, whereas more consolidated rocks have been identified higher than 500 MPa.

The Bulk modulus (K) map (Fig. 14. d) exhibits values between 907 MPa and 2978 MPa for the first layer, where high readings (greater than 500 MPa) in the middle and eastern sides reflect rigid, dense, and stiff materials, such as compacted soils or rocks. The central, northwestern and southwestern regions exhibit small values which correspond to softer substances such as loose formations. Higher readings (more than 500 MPa) are found for rigid deposits in the eastern and middle of the western side of the investigation area. The second layer, however, has values between 967 and 12,314 MPa. Low values in the research area indicate fair competence in the center, while high values in the northern and southern regions (Fig. 15. d.).

These moduli demonstrate increasing rigidity, stiffness, and density with depth, confirming a transition from soft surficial deposits to more consolidated subsurface materials.

#### Material competence scales

To further evaluate subsurface competency, several dimensionless indices were derived from the elastic parameters, including the material index (Mi), concentration index (Ci), stress ratio (Si), and density gradient (Di).

a. Material index (Mi).

**Fig. 16:**
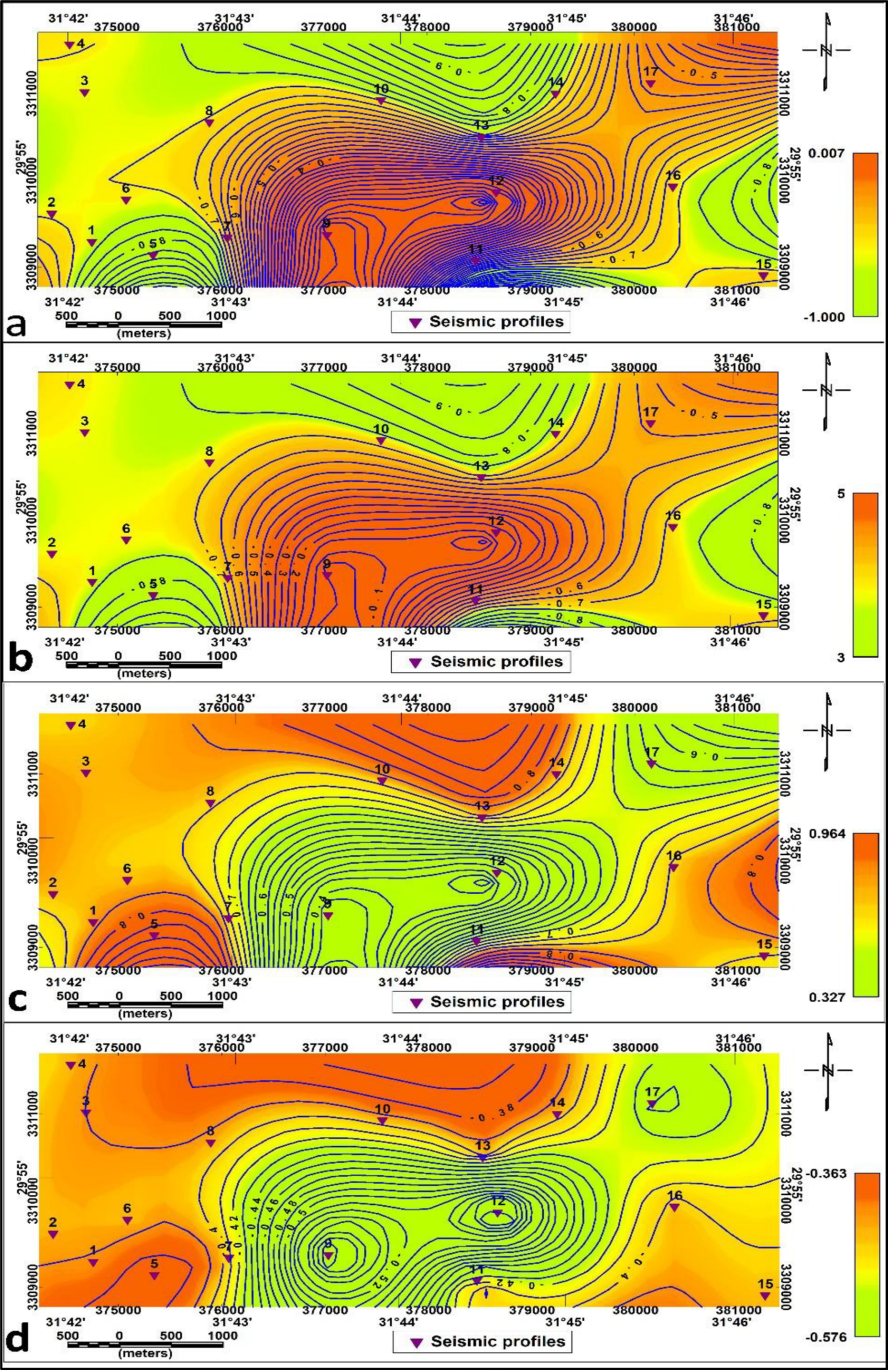
Competence scales lateral distribution for the first layer (**a**): Material index, (**b**): Concentration Index, (**C**): Stress Ratio, and (**d**): Density Gradient.

**Fig. 17:**
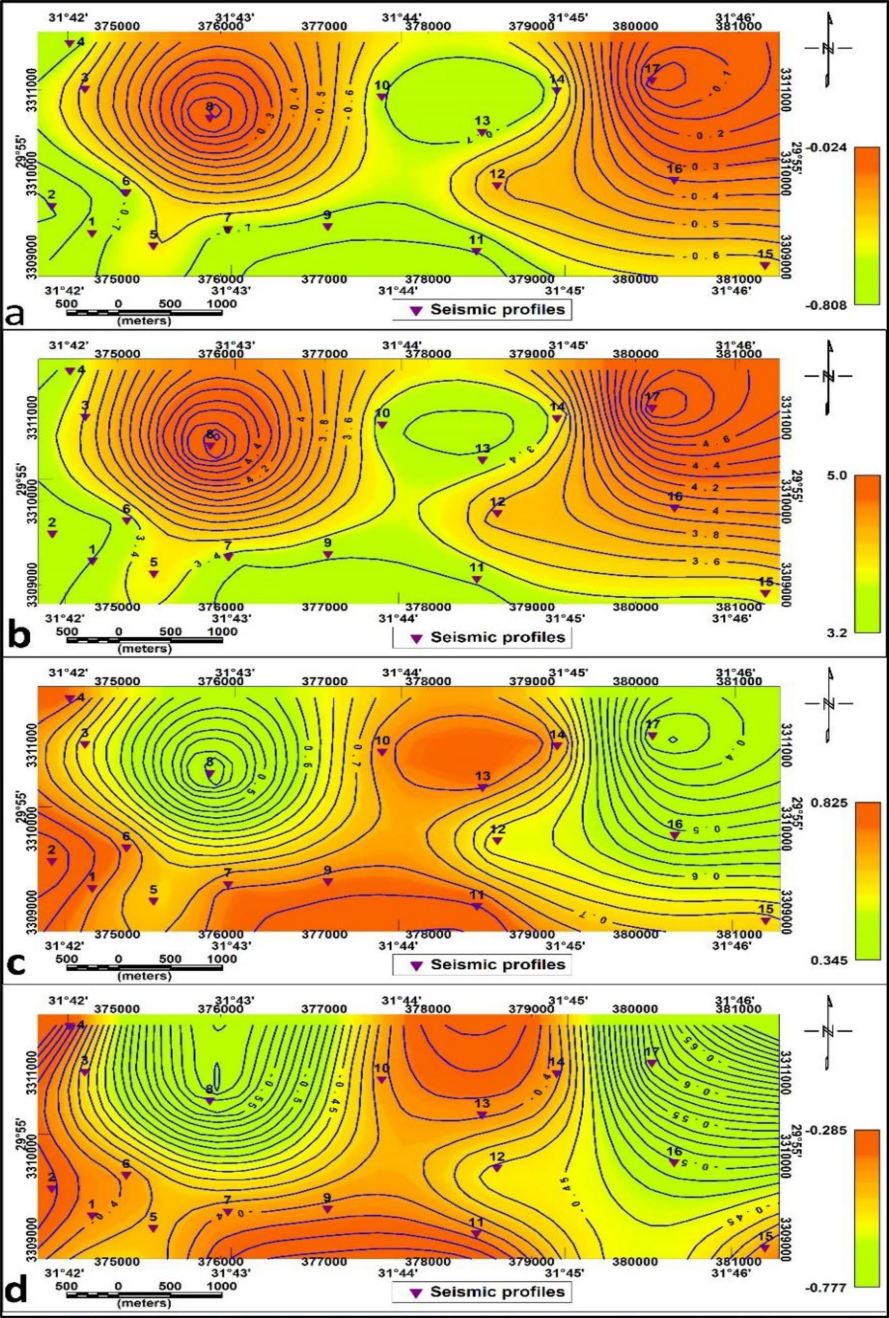
Competence scales lateral distribution for the second layer (**a**): Material index, (**b**): Concentration Index, (**C**): Stress Ratio, and (d): Density Gradient.

The Material Index (Mi) map (Fig. 16. a.) shows values between − 0.833 and 0.007. The values in the central area’s investigated section are medium-high for fairly to moderately competent materials and soft for medium. The range of values on the material index (Mi) second layer map is −0.822 to 0.02. The values range (−0.822 to −0.5), (−0.5 to 0) and (0- 0.02) exhibit 3 zones: slightly competent, fairly to moderately competent and competent materials respectively (Fig. 17. a.).

The Concentration Index (Ci) for the first layer reveals high values in the central and northeastern parts of the study area (Fig. 16. b.). Three zones are defined in the second layer based on concentration index values: the in-component to slightly competent zone (has low values), the fairly to moderately competent zone (have moderate values), and the competent zone (has relatively high values) in the northeastern side and around P8. (Fig. 17. b.)

The Stress Ratio (Si) map for the first layer (Fig. 16. c.) has readings that vary from 0.33 to 0.84. The central part of the research area has low values in the central and medium values related to soft and fairly to moderately competent, respectively. Readings on the second layer’s Stress Ratio (Si) map (Fig. 17. c.) range from 0.324 to 0.834.

Zones with low Si (≤ 0.35) indicate soft materials and low seismic velocity, moderate values (0.35–0.45) correspond to fairly competent zones, and high values (≥ 0.45) denote highly competent materials. These results provide valuable information for both geotechnical and seismic assessments.

These results provide important data relevant to technical and seismic studies that are consistent with standard values.

d. Density Gradient (Di):

The density gradient values for the first and the second layers are shown in Figs. 16.d and 17.d.

Depending on Mi, Ci and Di, the first layer study area is divided into slightly components in the south-central parts and fairly to moderate component materials in the other parts of the proposed area. In contrast, depending on the second layer, the research region was separated into fairly to moderate component materials in the outer parts of the proposed area and slightly components in the central parts.

Overall, these competence indices collectively reveal that the subsurface materials improve in stiffness and strength with depth, confirming the geophysical observations from elastic moduli.

#### Foundation bearing capacities

**Fig. 18:**
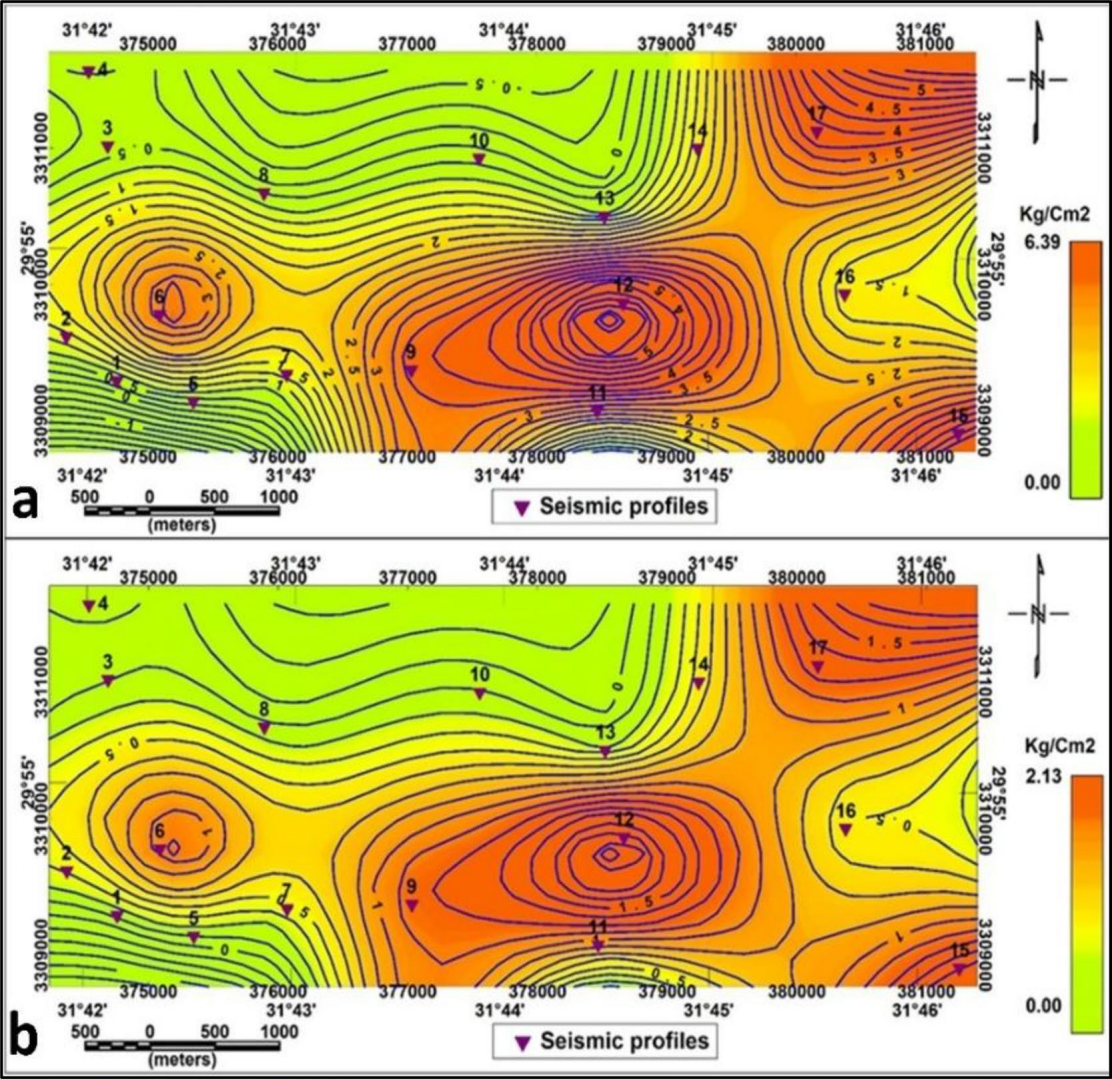
**a**- ultimate bearing capacity **b**- allowable bearing capacity for the first layer.

**Fig. 19:**
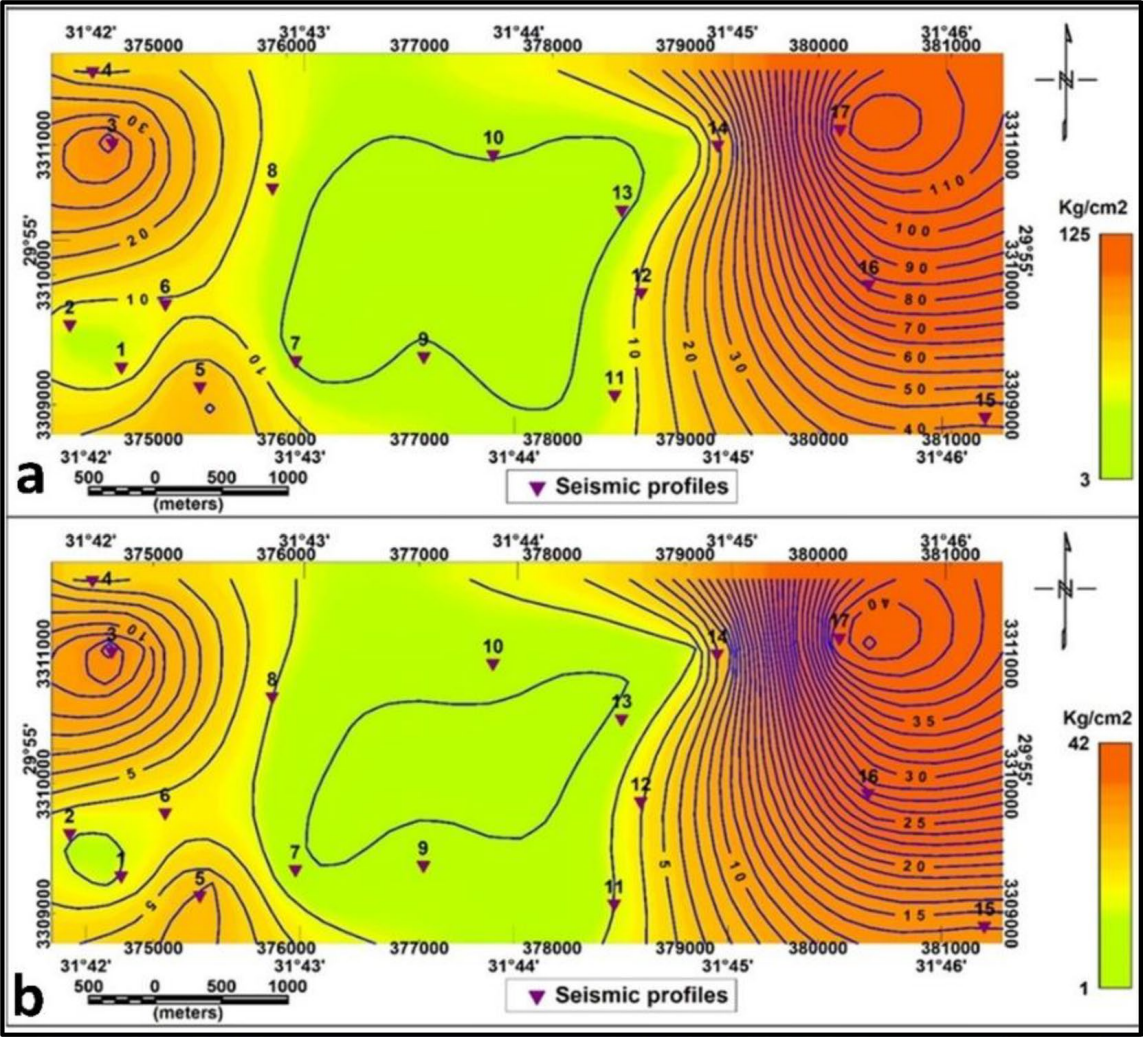
**a**- ultimate bearing capacity **b**- allowable bearing capacity for the second layer.

The variation of Ultimate bearing capacity (Qult) and Allowable bearing capacity (Qall) in the subsurface strata of the research region for the first layers is shown in Figs. 18. a and b respectively. The eastern, central and middle portions of the southern portion of the research region shows medium to high values. The northern and southwestern portions of the research region are where the ultimate and allowable bearing capacity values decrease to their lowest value. The second layer’s Qult and Qall contour maps (Fig. 19. a and b respectively) have quantities ranging from 2.6 to 128.3 and 1.4 to 42.8 respectively. In the eastern part, high values reflect dense, robust soils that can support significant weights, whereas in the center, minimal values indicate softer soils that need to be improved and the third is located in the western part of the research site, which the Qult and Qall have medium values.

#### Rock material classification

**Fig. 20:**
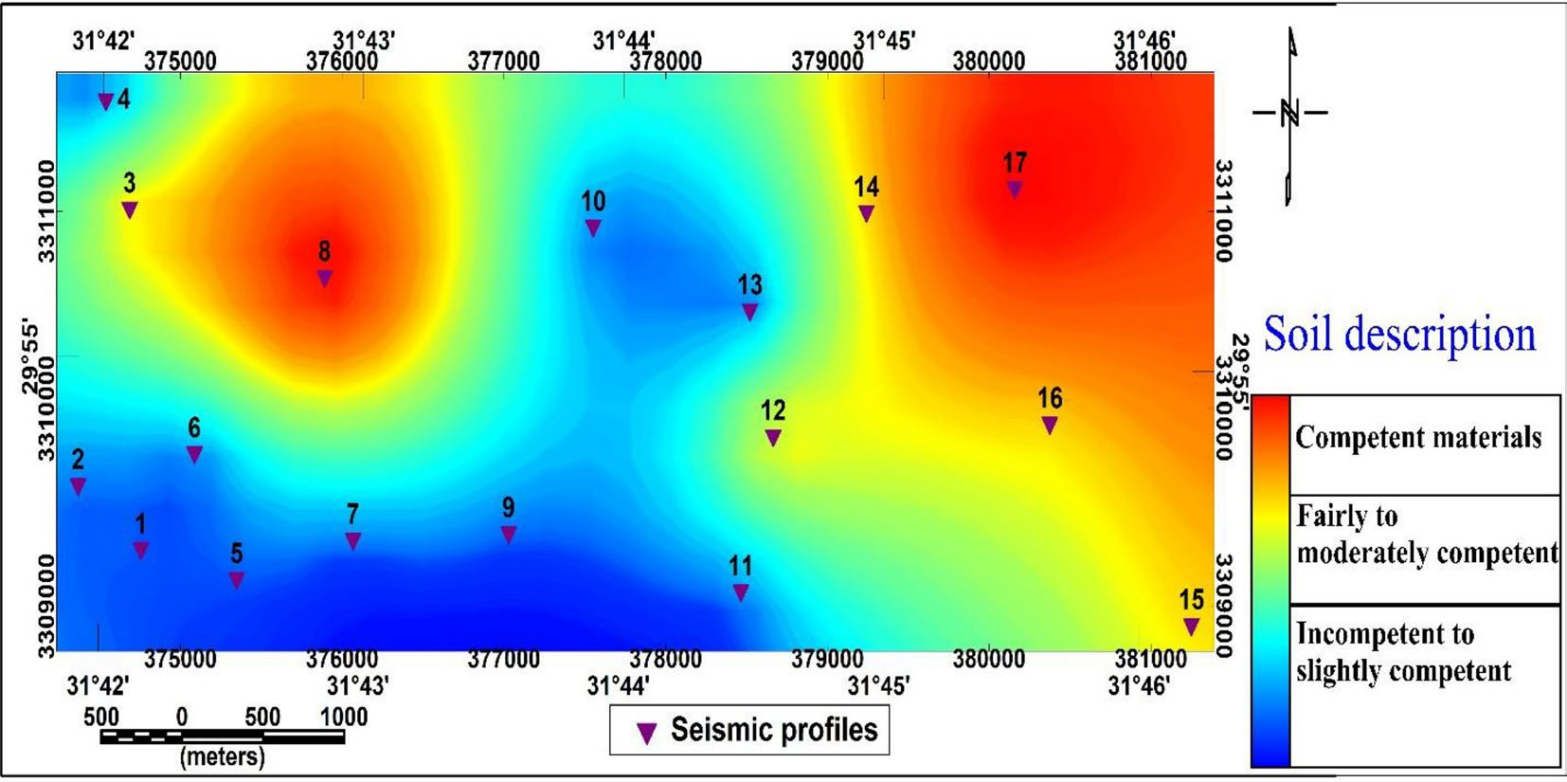
Soil competence zonation map Shows competent, moderately competent, and non-competent zones based on Poisson’s ratio and material index, indicating construction suitability across the study area.

For engineering purposes, the study area’s foundation rocks are categorized according to their Poisson’s ratio and material index^52^. This zonation map (Fig. 20) provides an overview of soil competence across the study site. The color gradient from blue to yellow to orange represents increasing material competence.

The surveyed area is classified into:

1-Zone (A): (competent material zone)

This zone, highlighted in orange, includes the northeastern sector and at p8. This zone is defined by competent materials suitable for construction and geotechnical applications.

2-Zone (B): (fairly to moderately competent)

This zone, marked by yellow coloring, represents a transitional area between incompetent to slightly competent zone, and a highly competent materials zone, reflecting fairly to moderately competent geotechnical conditions. This zone is located in the northeastern part nearest to the central at profiles 14,15, and 16 and also in the northwestern part at profile 3.

33-Zone (C): (non-competent material zone)

This zone is characterized by blue colors indicating incompetent to slightly competent soils in the southwestern and central portions of the study area. This zone occupies both the central and southeastern sectors of the study area.

## Conclusion

This study integrated electrical resistivity, seismic refraction, and MASW techniques to characterize the subsurface conditions of Egypt’s New Administrative Capital. The investigation identified a three-layer subsurface model and calculated key geotechnical parameters, which enabled a mechanical classification of the area into three competence zones (A, B, and C) with varying bearing capacities. The combined approach effectively delineated the subsurface structure and provided consistent correlations between resistivity, velocity, and geotechnical indices, supporting reliable assessments of foundation suitability.

The main contribution of this work lies in demonstrating the effectiveness of a multi-geophysical approach for high-resolution, non-invasive subsurface evaluation in rapidly developing urban areas. Despite its accuracy, the study is limited by the absence of direct borehole verification and depth constraints inherent to the applied methods.

For future work, the validated methodology itself is the primary contribution. We recommend its application to other new urban centers and arid regions in Egypt to build a comprehensive regional geotechnical database. Furthermore, translating these findings into a 3D subsurface model would be a powerful tool for city planners and digital engineering applications. This integrated approach provides a critical foundation for mitigating geotechnical risks in upcoming urban development.

## Data Availability

The datasets used and/or analyzed during the current study are available from the corresponding author on reasonable request.
